# Retraction for Huang et al., “Complete Genome Sequence of *Campylobacter coli* Bacteriophage CAM-P21”

**DOI:** 10.1128/MRA.00504-23

**Published:** 2023-11-28

**Authors:** Hung-Hsin Huang, Yu Zhang, Nanami Asoshima, Hoang Minh Duc, Jun Sato, Yoshimitsu Masuda, Ken-ichi Honjoh, Takahisa Miyamoto

## RETRACTION

Volume 10, no. 15, e00223-21, 2021, https://doi.org/10.1128/mra.00223-21. As scientific researchers, it is our responsibility to ensure that our data are accurate as well as representative. Resequencing to confirm the genome sequence of *Campylobacter coli* bacteriophage CAM-P21 presented in our article revealed that our originally published sequence is incorrect/incomplete. Therefore, a retraction of our announcement is necessary.

